# How Memory Structures Influence Distress and Recovery

**DOI:** 10.3389/fpsyt.2019.00500

**Published:** 2019-08-02

**Authors:** Alastair Dobbin, Sheila Ross

**Affiliations:** ^1^College of Medicine and Veterinary Medicine, University of Edinburgh, Edinburgh, United Kingdom; ^2^Foundation for Positive Mental Health, Edinburgh, United Kingdom

**Keywords:** memory, self-determination, emotional distress, recovery, depression

## Abstract

The treatment of emotional distress has stalled. There has been no improvement in depression outcomes for 30 years, and recent opinions have highlighted the lack of clear validity for the mechanisms involved in current models of complex psychological interventions. Emotional distress is a phenomenon that underpins a swathe of apparently diverse mental health conditions. Childhood trauma has been found to be endemic and common in those with poor psychological adjustment in adults. Executive processes cause a split between knowledge of an event and knowledge of its negative ongoing emotional effects so making integration of the memory of such events and resolution of its effects is a challenge. Recent research into the relationship between well-being and memory structures has converged with research into episodic memories and their key role in goal programming, giving an opportunity to describe a new model with plausible mechanisms for emotional distress, regulation, resilience, and recovery. Humans seek goals that fulfil their universal basic psychological need for self-determination, and the structures of memory networks drive behavioral algorithms that can satisfy or thwart that goal. We propose that a single underlying deficit in memory networks could underpin the psychopathology of emotional distress and that a new formulation of distress and recovery could drive a useful update on the mechanisms of psychological interventions with greater validity and utility for recovery and propose the **NEMESIS** (**Ne**gative **ME**morie**S** **I**ntegration **S**ystems) model.

## Introduction: Free Association Revisited

“Further investigation brought to light a second memory, which she denies having had in mind at the moment of Scene I. Nor is there any evidence to support its presence there. On two occasions, when she was a child of eight, she had gone into a shop to buy some sweets and the shopkeeper had grabbed at her genitals through her clothes.” (p. 410) (1) Project for a Scientific Psychology

Freud among others was an early exponent of seeking out important traumatic experiences by following links of associated chains of thought and/or memory—and created a process of **free association**, defined by a therapeutic environment where the client lay on a couch and described what came to mind spontaneously, like watching the view from a railway carriage. The use of visualization per se changes the mode in which we process memories in emotional distress (2, 3). In the case of Emma who suffered from agoraphobia and could not enter a shop alone, this uncovered a link between a relatively benign memory, when two men had laughed at her clothes in a shop, and the earlier, more traumatic memory of assault (see above). Free association of memories can create a bridge between, on the one side, sometimes apparently illogical behaviors and feelings which occur in certain situations, and on the other, otherwise inaccessible episodes of trauma memory. In analysis this process is sometimes called an affect bridge that takes a client from a current difficulty to a past event. Freud recognized that Emma’s earlier memory had transferred the cathartic energy of threat to the later experience. Most interestingly, Emma describes not having had the assault memory “in mind,” although she did *not* say that she had forgotten it.

Emma’s memory of assault is an episodic autobiographical memory, which is defined as a memory of a specific event that happened in the course of a day, which may be recalled under conditions of free association or come to mind spontaneously. Such memories are recognized to be activated by environmental cues acting outside of conscious awareness, actively shaping emotional responses and instrumental behavior (4, 5). Conway and Pleydell-Pearce (6) noted that episodic memories contain specific often highly visual memories of significant events sometimes with highly charged emotional aspects, and they postulated that, in order to prevent an overwhelming emotional experience, with its concomitant negative effects on mood, concentration, and global mental function, control or executive processes may deny conscious access to the memory, but the sympathetic autonomic response associated with the event memory will still be triggered by associative processes; indeed, paradoxically the person becomes “minutely sensitive to cues” (p. 261) (6). This causes the person to frequently experience a feeling of threat, with no conscious access to the originating memory, and to find himself, as noted by LeDoux, “in the throes of an emotional state for reasons you do not quite understand” (4, p. 203).

A particular category of episodic memories, **self-defining** memories—elicited by asking people to find a memory they feel defines them as a person in a positive or negative way—has been proposed to be particularly representative of the relationship of a person’s self-image to their key goals and motivations that continues to influence how they react to events and their interactions with the world (7). Such memories are frequently activated and have significant long-term effects (8). Singer and Salovey (7) proposed that self-defining memories may be “created” where people collapse a number of similar experiences into a single one, gaining access implicitly through the collapsed memory to a series of similar memories. Applying this principle retrospectively to the case of Emma, the assault incident with the sweet shop owner would be seen as collapsed into the incident with the young men laughing at her. Cases showing similar effects, that is earlier trauma subsumed in later non-trauma experiences, have also been noted specifically in cases of agoraphobia (9) while work on PTSD has suggested a similar twinned event structure, where PTSD originates in earlier trauma experiences compounded in terms of PTSD development by attachment issues (10).

Possibly clarifying these diverse phenomena is the finding that people instructed to recall a self-defining memory show an immediate increase in well-being if it is a positive memory, and recognize this effect, but conversely they do not recognize that negative self-defining memories have a negative effect on their well-being (11) (p510). This may be as a result of a negative self-defining (NegSDef) memory being excluded from consciousness, kept behind a firewall, while cues from the experience repeatedly trigger negative emotions [q.v. Ref. (6)] thus generalizing distress to other situations with associative similarities. Emma knew she had been assaulted, but until her analysis with Freud she had not recognized that this was what was upsetting her, or that it had any link to her agoraphobia, which had resulted in generalization of fear and shame to all shop environments.

## Action Algorithms, Goal Progress and Emotions

**Figure 1:**
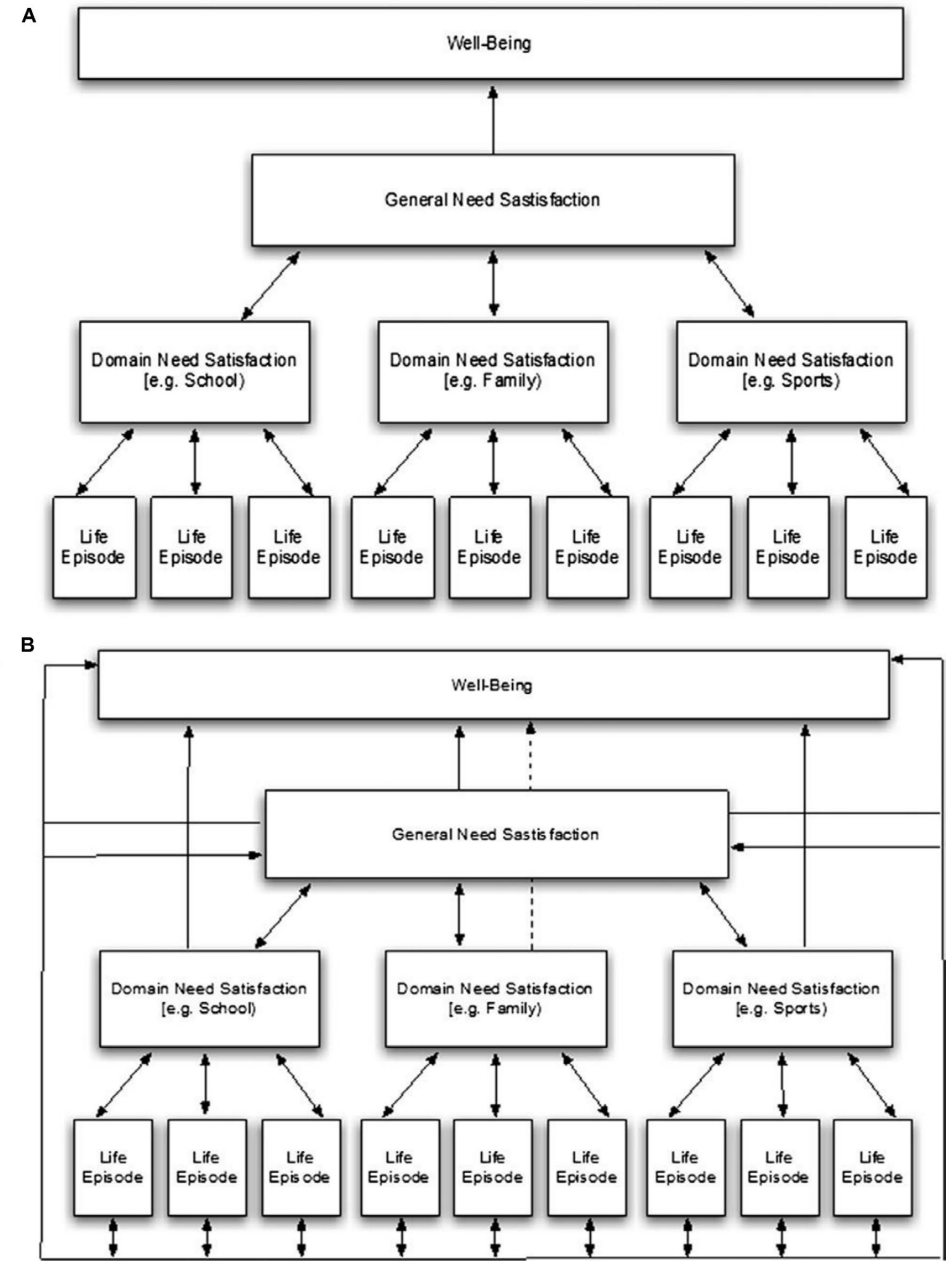
Hierarchical **(A)** and heterarchical **(B)** organization of interactions among three levels of experience and well-being. Reprinted from Journal of Research in Personality, Vol 47, Milyavskaya, M., Philippe, F., Koestner, R. Psychological need satisfaction across levels of experience: Their organization and contribution to general well-being, 41–51, Copyright (2013), reproduced with permission from Elsevier.

The treatment of emotional distress has stalled; there has been no improvement in the outcomes of depression treatment for 30 years (12), and it is felt that a better understanding of the mechanisms whereby distress is caused and therapies help is required (13). Recent years have seen an increase of interest in the structures and purposes of memories and the possible potential they hold for understanding and treating various forms of emotional distress. Many researchers and therapists in the field of memory, however, feel there are too many unknown factors in the structures, classifications, and processes of memory, so that the relationship of memory to emotional regulation and therapeutic processes cannot yet be understood (14–16). However, episodic self-defining memories, both positive and negative, have been shown to increase or decrease, respectively, short-term and long-term well-being and emotional health (11) which supports a model describing *in quantifiable terms* exactly such a relationship. It may seem counterintuitive that episodic memory could have such a large impact on long-term well-being, but a recent empirical analysis of this relationship has shown that, rather than a hierarchical model of well-being where episodic memory has a bottom up effect on overall well-being, a heterarchical model is preferred, with episodic memory affecting well-being on many different levels, exerting an omnidirectional effect, simultaneously bottom up and top down (17). In this way even a single episodic memory (a life episode) can simultaneously affect well-being, general need satisfaction and domain need satisfaction, thereby occupying a key and central role in well-being and emotional distress (see **Figure 1**, model B).

## What Are Memories for?

Many researchers have concluded that episodic memories have a particular relationship to goal attainment, providing information that will assist in smooth goal progress (18, 19). Episodic memories are proposed to be retained long term only if they have a goal-directed purpose; thus, the lack of early childhood memories may be related to a change of goals from childhood (affection and nurturing) to adolescence (independence, mastery, and intimacy) the latter continuing to be useful throughout adult life (6, 20) with the childhood goals becoming redundant in helping integration into the adolescent/adult world. Episodic memories have been found to have a common structure (21) with boundary conditions that start with information of an action and end with details of the outcome. It has been suggested that the highly visual nature of episodic memories allows the storage of a large amount of action-relevant information, allowing remembering in a way that is critical to focussed and fluent everyday cognition and action, strengthening the view that episodic memories are created and structured to maximize goal progression (18, 22). Brain injury or degenerative processes that compromise access to episodic memories are extremely disabling for effective action and future planning; these findings all point to a common conclusion, that episodic memories form the basis of situational-relevant algorithms for action geared towards goal progress (15, 21, 23). In a situation where goal progress slows, current algorithmic memories can be retrieved and compared with other episodic memories which might be more effective in goal progress attainment in that situation (14, 24). This process might be progressed in a reflective processing mode, a state of restful waking or daydreaming, or possibly one of many types of manifestations of a pause in external attentional engagement which triggers a different form of mental processing, for instance, in napping, which is recognized to improve procedural and declarative memory (25, 26), or visualized recall, which can change the emotional trajectory of memories (2). It is particularly notable that activity in the default network, which is active during restful waking (daydreaming) and dreaming, is negatively correlated with activity in attentional networks and is active in studies of recalling the past and imagining the future during which apparently random memories may come to mind (27–30).

The purpose of emotion may appear to be complex but can be reduced to one simple principle by applying parsimony; emotion is principally a mechanism to signal the current state of goal progress, with negative emotions signalling frustration of progress (and a need to revise) and positive emotions signalling smooth progress and encouraging engagement with new goals (31–33). This means that emotional distress can be reframed as a state of diminished goal progress. However, avoidance of distress from threats, i.e., fear, can also be a goal, the goal being effective emotional regulation (32). Progress towards this goal maintains positive emotions (relief). The observed effect of a negative self-defining memory, which is often unrecognized as a source of distress (q.v. 11), may be a drop in positive and rise in negative emotions (34) if memory is not integrated; see *Network Memories and Emotional Regulation*.

This focus on the nature and purpose of memory to guide and refine behavioral strategies to maintain goal progress and assist problem solving enables a broader view of the interaction of memory with emotional distress and regulation. Predictive coding theory (35–37) outlines how the higher centers involved in planning (i.e., cerebral cortex) predict the outcome of an event/situation based on past experience(s) which underpins an action algorithm to follow in that situation. This algorithm is based on a model of hidden causality (hidden from explicit understanding), which generates a top down prediction of what the sensory feedback from the algorithm action will look and feel like. This prediction descends to the peripheral sense organs and at each level matches the prediction to the actual data ascending bottom up from all the sensory channels, including autonomic/emotional information, feeding back through the vagus and the spinothalamic tract as the action unfolds in real time. If the expected goal progress is lacking, this will change the sensory feedback (emotion) signalling a prediction error creating a surge of positive cerebral electrical activity, free energy, which is visible as a positive deflection at 300 ms on event-related potential studies (38), occurring in parallel with a sympathetic response, increased skin conductance, and heart rate (39), all part of a process that focuses attention on the unsuccessful algorithm and drives a revision of the components (15, 19) to help current problem solving.

These two approaches to goal functioning and successful/unsuccessful episodic memory algorithms converge on observations of the nature of internal examination of past events. Rumination represents a state of reiterative circular thinking of the past focussing on the outcomes of events, and there are two aspects to such thinking, moody pondering (brooding) which leads to depression and self-reflection which is beneficial and adaptive and focuses more on problem solving (40). Brooding is associated with increased activity in the default network particularly in major depressive disorder which could signal a fruitless search for more functional goal fulfilling episodic memories (41). The default network has been linked to reflection on memories self-identified as key stressful episodes in a relationship to a significant other (similar to a negative self-defining memory), a process recognized to be of key importance in negotiating the social environment (42). Problem solving therapy focuses on visualizing the steps to achieve resolution, which would implicitly involve past memories and the default network.

## Positive Psychology and Resilience

During the early years of the new millennium the Positive Psychology movement (PPM) shifted the focus of research away from the pathways, functional anatomies, and outcomes of *negative* emotions and schemas, towards a focus on the pathways and outcomes of *positive* emotions. An early research focus was resilience, how to define and measure it, and the mechanisms whereby it helps people through distress. Research had previously shown that the defining emotional characteristic of depression was a lack of positive emotions (43). Early findings of the PPM were that resilience is represented by an ability to more rapidly recover psychologically and physiologically from threat. After the traumatic incident of 9/11 in New York, resilient American people experienced psychological growth while non-resilient people became depressed, and these outcomes were fully mediated by access to positive emotions (44). In empirical studies physiological resilience (represented by the speed of diminishing the sympathetic nervous system activity) was also found to be fully mediated by the experience of positive emotions (45), which was later found to be mediated through central autonomic control nuclei (46). This supports an oppositional-inhibitory model of autonomic function in which parasympathetic and sympathetic activity, correlating with the behavioral expression of positive and negative emotions respectively, inhibit each other at multiple spinal and cortical levels (47–49) as well as *via* the dense connectivity between ascending, descending, and modulatory networks and midbrain nuclei particularly the peri-aqueductal gray (50). Positive emotions have been shown to have multiple beneficial effects on the brain and body, enabling all sorts of approach behaviors through widespread cortical neuromodulation (51, 52) while simultaneously diminishing the expression of negative emotions (53) and encouraging the seeking of additional approach goals (32, 33) making increasing positive emotions a potentially useful therapeutic target.

## Network Memories and Emotional Regulation

In recent years a research group in Montreal, based initially at McGill University and latterly in the Laboratory for Research on Emotions and Representations (ELABORER) in the Department of Psychology at the University of Quebec in Montreal, headed by Prof. Frederick Philippe, has established a framework of research into memory which is showing promise in uncovering the mechanisms underpinning emotional distress and the factors that protect against this. Recognizing the research findings on the psychological effects of 9/11 (44), they argued that the positive emotions that mediate resilience must be self-generated “produced—in an explicit or implicit fashion—by the person’s internal resources” (p. 140) (54), and if this process could be measured it could be linked to future mental health and well-being outcomes, leading to a delineation of memory-related emotional regulation processes as suggested in Singer and Savoley (7). They proposed that the emotional and behavioral outcome of challenging situations might be governed by the simultaneous activation of a number of different episodic memories each with potential positive or negative effects which might temper or augment access to positive emotions (54). They looked for negative memory associations with stressful situations but also positive episodic memory associations arising from the same situation that might assist a person to attain their optimal situational function and increase their well-being. They found as before (42) that the key factor governing such an outcome was resilience in the form of access to positive emotions, but crucially, they also found that the key factor mediating access to positive emotions was the structure of memory networks (54)—which their subsequent research has systematically elucidated. They have also shown that the level of goal progress in these memory networks is directly related to well-being, linking the network structure to one of the accepted principal functions of episodic memory (55). They have established through empirical research a series of important observations.

Episodic memories are nested in **networks** of other episodic memories that share similar events, themes, and emotions, i.e., social or recreational events; themes of loss, power, or aggression; emotions such as sadness, happiness, shame, or anxiety (34). When one memory is activated by a challenging situation (**the main memory**) other memories in the network (**network memories**) will also be activated. These will be the memories that resonate most strongly with the main memory, and the combination forms a relatively stable **memory network** (55). Although not usually coalescing in awareness into fully formed memories (6), for the purposes of research such memories can be uncovered by free association, and this process has underpinned the rigorous empirical work of the ELABORER programme of research. To find a negative self-defining (NegSDef) memory, people are asked online to “describe in detail (like telling a story) a NEGATIVE personal memory of an event/moment…. that is significant (important) for you …. (which)… should reflect your identity or what you are as a person” (p. 4) (34), and for the network memories they are asked to describe any other events that spontaneously come to mind after recalling the main memory. Understandably the researchers recognized the link with the process of free association (54).

The network memories of the memory network can each be classified by their ability to help the individual achieve goal progress in the situation that has activated the main memory. The ELABORER group explored several models which linked memories to well-being and found that the level of **self-determination** expressed as the level of needs satisfaction experienced in memories provided the most robust link between an episodic memory’s components and well-being (55). Self-determination is a quality which underpins an individual’s sense of well-being and of purpose and meaning (56) and is correlated with the level of satisfaction of the basic psychological needs/goals of all people across nations and cultures (57). The components ELABORER use to measure needs satisfaction in memories are **Autonomy**—an ability to act and think independently, **Competence**—having a useful skill set to bring to bear, and **Relatedness**—a feeling of connectedness to others. Each component is scored by two items, one positive, one negative, on a seven-point Likert scale from *strongly disagree* (−3) to *strongly agree* (+3) with 0 representing *neither agree nor disagree*. Autonomy is scored by the positive statement “I felt free to do what I wanted” and the negative statement “I felt obliged to do what others wanted” (55), the latter reverse scored. For the other two components the positive statements are “I felt I had the skills in that situation” (competence) and “I felt close to others in this memory” (relatedness) (55). The items (six in all) are averaged together to form an index of needs satisfaction for each network memory, separately, resulting in each network memory being either **needs satisfying (NS)** if the index score is >0, or **needs thwarting (NT)** if the index score is ≤ 0, a dichotomous outcome (55). The first three network memories accessed have been found to contribute to the overall self-determining outcome when the memory network (main memory and network memories) is activated (34, 55). Beyond these three memories any subsequent network memories recalled have no additional predictive power of the outcome of activating the memory network. The influence of these three network memories is described by the **memory network integration score**, which is the sum of NS and NT effects, the number of NS network memories minus the number of NT network memories. In the same way as the outcome of each individual network memory is dichotomous, needs satisfying or thwarting, so the overall outcome of the memory network integration score is also dichotomous, either needs satisfying—increasing well-being when activated, or needs thwarting—decreasing well-being on activation. The influence of the main memory is completely dependent on its memory network integration score, which, if positive (NS), entirely mitigates the stress of the challenging situation that gave rise to the main memory, leading to psychological growth, the experience of positive emotions, and long-term well-being (11).

**Glossary Terms**

**Episodic memory:** an autobiographical memory of an event that spans a single day.

**Main memory:** the first memory that comes to mind when a current event activates an episodic memory.

**Network memory:** any memory that comes to mind after the main memory. The first three network memories contribute to the memory network integration score.

**Memory network:** a unit composed of a main memory and its network memories.

**Self-defining (SD) memory** (may be positive or negative—NegSDef): a memory that comes to mind when people are asked to find something that defines them in a positive or negative way. Such memories appear to tap into fundamental perceptions of self and collapse (combine together) recent and past trauma memory.

**Self-determination**: The sense of well-being that comes from moving towards goals, composed of a sense of autonomy, competence and relatedness, and purpose and meaning. Self-determination drives motivation and increases well-being.

**Needs-satisfying memory:** one in which people remember having a sense of autonomy, competency, and relatedness at the time of the event.

**Needs-thwarting memory:** one in which people remember not having a sense of autonomy, competency, and relatedness at the time of the event.

**Memory network integration:** the needs satisfaction level of the combined networked memories (not including the main memory). Result may be positive or negative; if negative, decreasing well-being.

Thus, the research of the ELABORER group, through detailed examination and analysis and comparison of various models of the mechanisms whereby episodic memory may play a pivotal and measurable role in emotional regulation, has converged with basic memory research into the structure and purpose of episodic memory. This strengthens the conclusion that such mechanisms exist to find a supportive structure of network episodic memories that can create the algorithms that support the fundamental and universal psychological goals of autonomy, competence, and relatedness, with both research strands strengthening the others’ findings.

The extensive research of the ELABORER group has shown that a negative self-defining (NegSDef) main memory, with a negative network integration score (i.e., non-integrated), is repeatedly activated outside consciousness by environmental cues, as described in Conway and Pleydell-Pearce (6), and lowers well-being in the short term and also the long term (11, 58). Integration is proposed as a process of acceptance of self, represented by a NegSDef main memory with a positive network integration score, which results in a person *not* being repeatedly traumatized by environmental cues of unrecognized origins but demonstrating their resilience *via* access to sufficient needs-satisfying network memories to create a positive future action visualization driven by positive emotions when the main memory is activated; or to put it another way, the algorithm created by the network memories ensures a positive self-defining outcome. The NS or NT effects of NegSDef memory network with a positive or negative network integration score respectively on well-being goal progress and positive emotions is above and beyond the effect of age, gender, personality traits (the big 5), and psychological symptoms and has been shown to maintain its predictive effect up to at least a year later (11).

**Figure 2:**
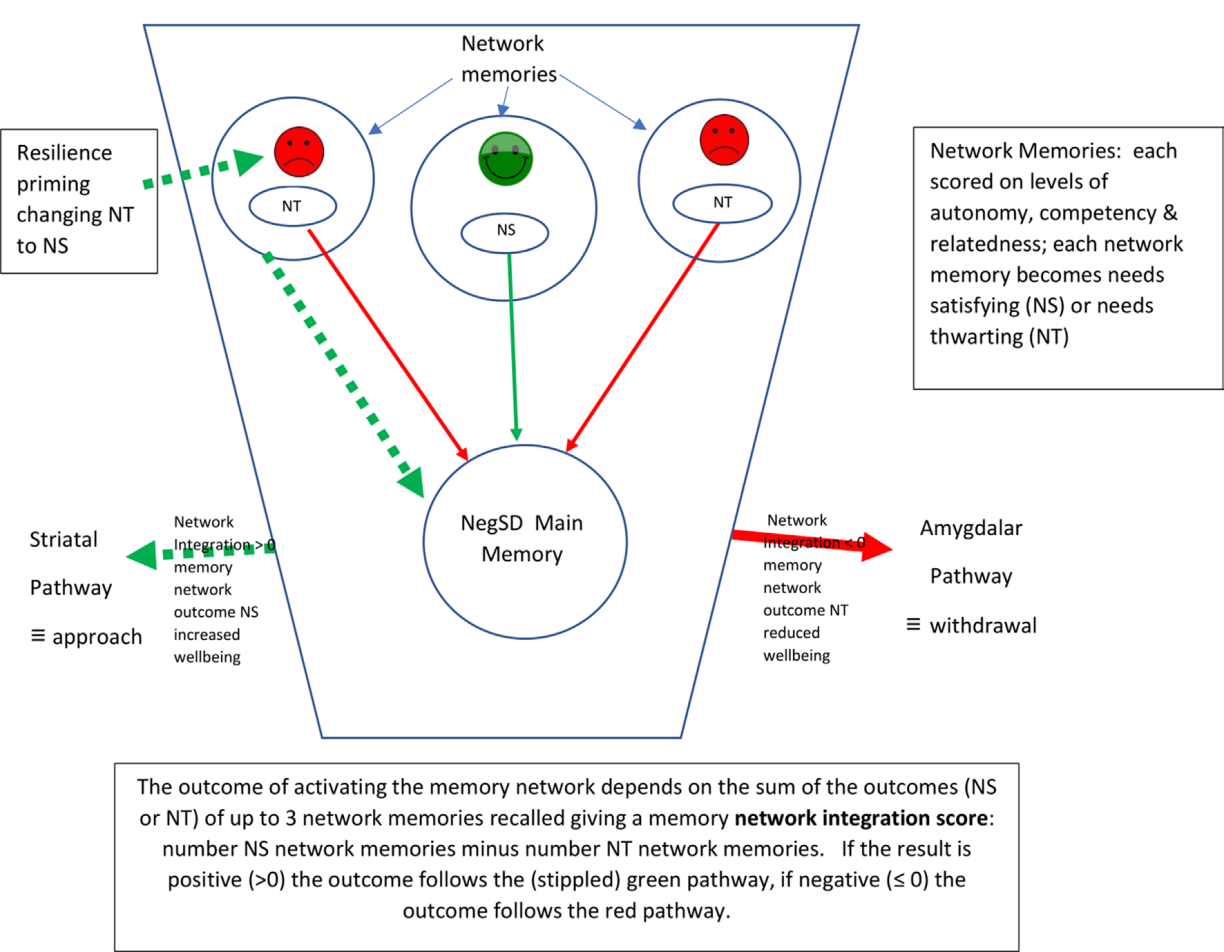
Diagram illustrating Memory Network structure and activation pathways.

For a figure illustrating the relationships of memory network pathways see **Figure 2**.

## Establishing a Mechanism for Resilience and Recovery: The Impact of Memory Networks on Resilience and Well-Being

From a point of view of devising any kind of therapeutic use for this memory-related emotional regulation mechanism, the key question is this: as we recognize that it is the negative self-defining memories that cause long term lowering of well-being and thwarting access to positive emotions, can a NegSDef memory with a needs-thwarting network have its memory network changed to needs satisfying?

We (AD and SR) had previously observed that a particular form of positive resilience priming, an implicit instantiation of an emotional growth model with similar principles to Hong et al. (59) (study 3), could nudge those in a state of emotional distress towards a reappraisal of past dysfunctional memories, resulting in an increase in quality of life and reduction in depression (60). This led to us collaborating with ELABORER in a randomized experimental study of a group of university students to test the effect of a 10-min resilience priming audio (the intervention group) on the self-determination levels of a negative self-defining (NegSDef) main memory and its network memories when compared to an active relaxation control group and a neutral music control (34). In this study 89 psychology students studying at a Canadian University (70 females, 13 males, 6 lost to follow up, mean age 20.30 years, SD = 1.57 years) were recruited. The RCT was split into two phases. During phase one psychological adjustment was measured using a composite scale of six individual scales measuring depression, anxiety, stress, and well-being; previously validated by ELABORER, Cronbach’s alpha coefficient was 0.90 for this index. Students were then asked to recall a NegSDef main memory, and free associate up to three network memories. Each network memory was assessed for NS (needs satisfaction) or NT (needs thwarting) outcomes, and the memory network integration score was calculated. Phase two took place a week later and involved participants recalling their main negative self-defining memory prior to randomization to one of three 10-min audio conditions: resilience priming, music, or relaxation. Positive emotion was measured both pre-recall (T1) and post-intervention (T2). Additionally, NS and NT were measured for the main memory and each network memory at T2. ANCOVA analysis using the conditions as the between factor was performed on positive emotion at T2 with positive emotion at T1 as covariate *F*(2,79) = 3.11, *p* = 0.05; the integration score at T2 with integration at T1 as a covariate *F*(2,79) = 3.24, *p* < 0.05; and the need thwarting ratings of the negative self-defining memory [*F*(2,79) = 0.06 ns]. Where significant main effects were found, planned orthogonal contrasts were carried out and regression analysis was performed.

Analysis revealed at baseline, in all groups, that there was a positive correlation between the level of psychological adjustment, the NegSDef memory network integration score, and the level of positive emotions. Controlling for baseline positive emotions and network integration, the resilience intervention group showed a significantly greater increase in the mean memory network integration score (+1.48 SE 0.28), compared to the control groups (+0.54 SE 0.38). This is equivalent to an increase of about one needs-satisfying memory more than the control in those listening to the resilience intervention. Given that the maximum possible integration score is +3 (range −3 to + 3), this represents a significant shift from a negative memory network integration score in the memory networks of students who were experiencing needs thwarting in their memory network at baseline to a positive score, a needs-satisfying memory network, integrating and accepting a previously traumatizing main memory. Supporting this thesis, the resilience intervention group also showed a moderately greater average rise in positive emotions post intervention, with analysis revealing that this was due to a large rise in those who started with a low level of positive emotions at baseline (with low psychological adjustment), while those starting at a high level (well adjusted) had no change. This suggests that those with good psychological adjustment were already using a general strategy of memory network integration for emotional regulation, having already in place enough positive self-defining network memories to achieve positive network integration, such that they were able to “recruit positive emotions… (from such network memories) … and recover to their baseline level after recalling a negative event” (p. 7) (34) as a form of emotional regulation. Taking a broader outlook, they had managed to change the perception of a past event, their main memory, from causing goal frustration to goal satisfaction of their fundamental psychological needs by increasing by approximately one the number of needs-satisfying network memories associated with the main memory. The main memory was not changed in our study, but something changed in the network memories, which enabled a new perspective of the self, an integration or acceptance of the dysfunctional main memory. We do not currently know what the exact change was in the network memories, whether one or all were different memories or were reappraised versions of existing network memories; given the emphasis on reappraisal in the priming script, we would think the latter. Further research should reveal this.

This study taken in context with the other research from the ELABORER group points to a plausible mechanism for emotional regulation in the emotionally well-adjusted: positive network integration of a NegSDef memory network. It also points to a mechanism for emotional distress in those with low psychological adjustment, i.e., non-integration of a NegSDef memory network, and a possible mechanism for bringing about recovery from the ongoing damaging influence of this non-integrated memory network, i.e., by using resilience priming to change one of the network memories. This may explain the results of an earlier study of the effects of incorporating such a resilience priming intervention (which includes emotional stabilization as a safety net to avoid emotional flooding) in depression therapy which found increased quality of life compared to cCBT in depression, particularly severe depression (60). The neuroanatomical and psychological correlates of default network function with its focus on past memory and future action visualization are perhaps involved in enabling a timely propitious implicit nudging towards a more beneficial memory network which those not in a state of emotional distress are able to automatically employ. Thus, we have a potential therapy that strengthens the mechanism that regulates emotions.

## Resilience and Well-Being Revisited

We see this work as underpinning a new model and understanding of emotional distress, regulation, recovery, and resilience, with clear and measurable mechanisms based on empirical science. With this new knowledge of the source of positive emotions that mediate resilience, i.e., episodic memory networks, we propose a new model of recovery and resilience—**NEMESIS** (**Ne**gative **Me**morie**s** **I**ntegration **S**ystems). In this model emotional distress is generated by a non-integrated, needs thwarting (NT) negative self-defining (NegSDef) memory network (see **Figure 2**) constantly thwarting basic psychological goals outside awareness, repeatedly activated by cued challenging situations. Recovery from distress is by the integration of the NegSDef memory through changes to its network memories.

Resilience protects against withdrawal, speeds recovery from stress, promotes approach-based behaviors over withdrawal, reduces vulnerability to depression, and is mediated by access to positive emotions (44, 45). Positive emotions are now recognized to be mediated by goal progress in universal self-determining psychological needs derived from positive episodic memory networks (11, 54, 55), and our experimental study (34) has validated that finding. The questions around the validity and reliability of the concepts of the ICD and DSM (61) calls into question the epistemological basis of current classifications of the causes of emotional distress and hampers progress on recovery programmes. NEMESIS is a transdiagnostic model, reflecting the concerns of the Research Domain Criteria (62) and the Lancet Commission on Psychiatry (13), both of which suggest the lack of progress in mental health outcomes in the last 30 years is due to a lack of validated models for emotional distress and psychological and psychiatric treatments. We propose that there are many different pathways which express emotional distress: depression, anxiety, PTSD, phobic disorders, borderline personality disorders, etc., but that all such distress, no matter what the expression, may originate in experiences held as non-integrated NegSDef memories. The NEMESIS model is underpinned by clear mechanisms of distress regulation and recovery which, in bringing together two converging strands of complementary basic memory research, may be more epistemologically valid than the current reification of the expressions of distress, which are descriptors of symptoms rather than conditions with clearly delineated mechanisms (61), and may fulfil the need for clarification of mechanisms for distress and recovery (12). Since the findings and ongoing revelations of the Adverse Childhood Experiences (ACEs) study in the US have revealed the astonishingly high levels of difficult early experiences in a normal population (63), it seems entirely credible that unresolved memory structures could be exerting the major effect on many manifestations of subsequent mental disorders.

## Limitations

This theory has only been tested in the short term by applying resilience priming and measuring the outcomes. We have yet to establish what makes the difference in the memory network: is it a different perspective on one of the network memories, moving it from thwarting to satisfying, or is it finding one or more completely new memories for the network and in the process increasing the number of needs-satisfying memories? This may yield more insights into memory processes that cause the observed effect of changing the outcome measurably for the better, and a greater understanding of the mechanics of recovery will have economic as well as clinical benefits. Clearly, more studies are required into the therapeutic use of such methods, particularly RCTs, but with careful consideration of the negative effect of expectation of failure in those receiving TAU (a nocebo effect), treatments must always be compared to active placebos (control groups with a recognized therapy) as well as TAU.

## Conclusion

It has been demonstrated that in any challenging situation the sense of autonomy, competence, and relatedness held in associated memory networks will drive or thwart access to positive emotions which impacts positively or negatively, respectively, on well-being and behavioral strategies, driving approach or withdrawal strategies. Non-integration of a negative self-defining memory will cause long-term withdrawal and lowered well-being without conscious awareness of the cause. The NEMESIS model addresses the need for a better understanding of mechanisms that underpin emotional distress and recovery. It is a model that integrates recent basic memory research into the nature and structures of memory-driven goal progress with the work of the ELABORER group to provide a structure of distress and recovery. By so doing it may promise better treatment outcomes, which have not improved for 30 years, and merits a diversion of research funds into this area. Existing networks of therapists could easily adopt this model, and there are existing cost-effective treatments that incorporate these mechanisms (60).

## Data Availability

All datasets for this study are included in the manuscript and/or the supplementary files.

## Author Contributions

All authors listed have made substantial, direct, and intellectual contribution to the work and approved it for publication.

## Funding

This study has been funded by the Foundation for Positive Mental Health (Scottish Charity no SC 041132). Publication is financed by the College of Medicine and Veterinary Medicine at Edinburgh University.

## Conflict of Interest Statement

AD and SR are shareholders in the company Positive Rewards Ltd which holds the patent on the recording used in the study Philippe et al. (2017). They are also directors of a Charity The Foundation for Positive Mental Health, a charity registered in Scotland which is dedicated to providing a mental health recovery programme, Positive Mental Training, to be free at the point of delivery across the National Health Service in the UK and to promote research into the use of this programme and its positive psychology principles across Society generally.
